# Supplementary material for: Complex‐centric proteome profiling by SEC‐SWATH‐MS
Source: Mol Syst Biol. 2019 Jan 14;15(1):e8438. doi: 10.15252/msb.20188438 (PMC6346213; doi:10.15252/msb.20188438)
Supplement: Supplementary file 7 — Dataset EV6 [file MSB-15-e8438-s007.zip › feature_plots_bioplex/Q9H410.pdf]

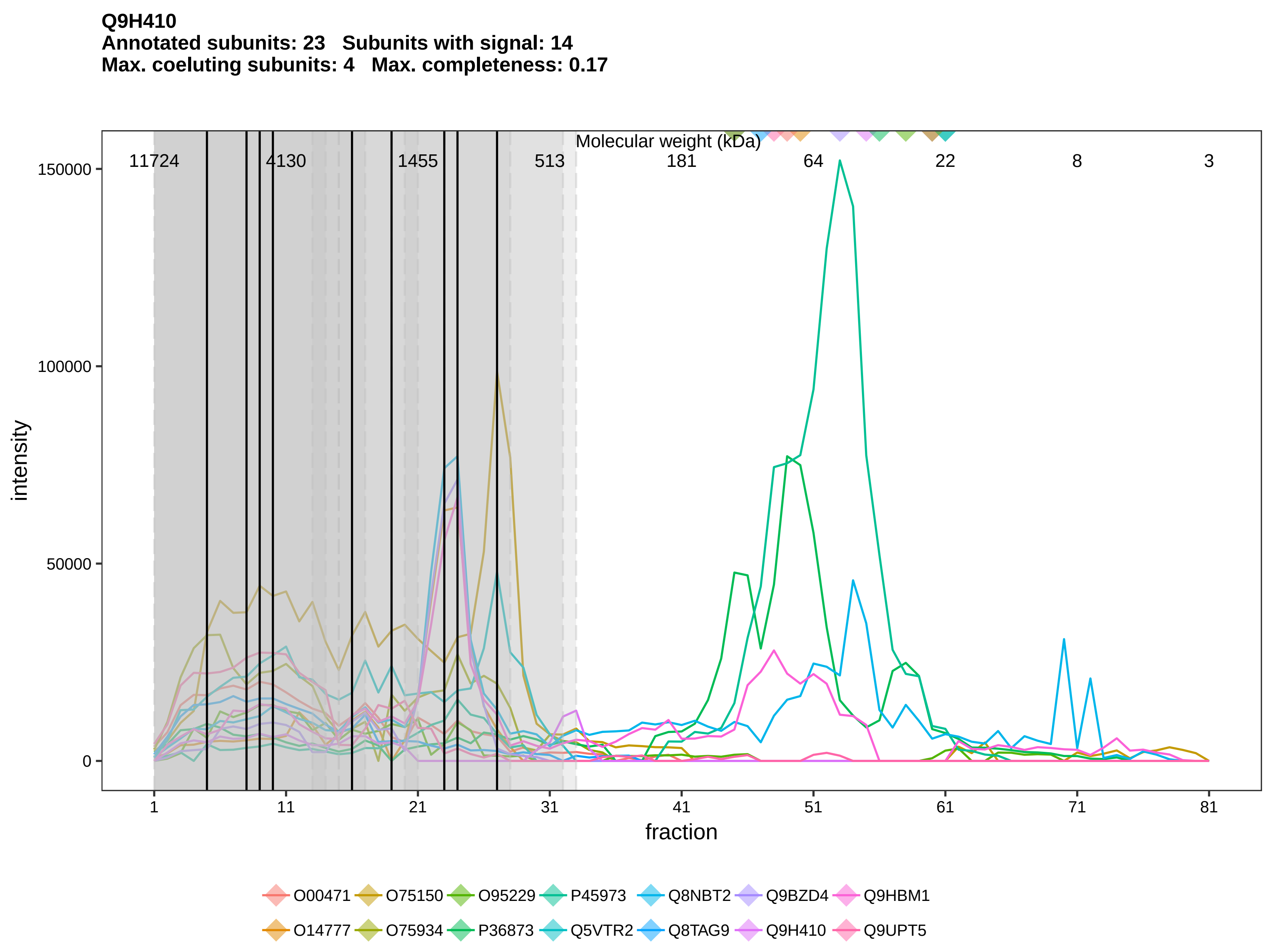

Q9H410
Annotated subunits: 23   Subunits with signal: 14
Max. coeluting subunits: 4   Max. completeness: 0.17
Molecular weight (kDa)
11724
4130
1455
513
181
64
22
8
3
150000
100000
50000
0
intensity
1
11
21
31
41
51
61
71
81
fraction
O00471
O75150
O95229
P45973
Q8NBT2
Q9BZD4
Q9HBM1
O14777
O75934
P36873
Q5VTR2
Q8TAG9
Q9H410
Q9UPT5
